# Molecular Analysis of a Short-term Model of β-Glucans-Trained Immunity Highlights the Accessory Contribution of GM-CSF in Priming Mouse Macrophages Response

**DOI:** 10.3389/fimmu.2017.01089

**Published:** 2017-09-11

**Authors:** Sarah Walachowski, Guillaume Tabouret, Marion Fabre, Gilles Foucras

**Affiliations:** ^1^Université de Toulouse, INRA, INP, ENVT, IHAP, Toulouse, France

**Keywords:** β-glucans, macrophage, trained immunity, Dectin-1, GM-CSF

## Abstract

β-Glucans (BGs) are glucose polymers present in the fungal cell wall (CW) and, as such, are recognized by innate immune cells as microbial-associated pattern through Dectin-1 receptor. Recent studies have highlighted the ability of the pathogenic yeast *Candida albicans* or its CW-derived β(1,3) (1,6)-glucans to increase human monocytes cytokine secretion upon secondary stimulation, a phenomenon now referred as immune training. This ability of monocytes programming confers BGs an undeniable immunotherapeutic potential. Our objective was to determine whether BGs from *Saccharomyces cerevisiae*, a non-pathogenic yeast, are endowed with such a property. For this purpose, we have developed a short-term training model based on lipopolysaccharide re-stimulation of mouse bone marrow-derived macrophages primed with *S. cerevisiae* BGs. Through a transcriptome analysis, we demonstrated that BGs induced a specific gene expression signature involving the PI3K/AKT signaling pathway as in human monocytes. Moreover, we showed that over-expression of *Csf2* (that encodes for GM-CSF) was a Dectin-1-dependent feature of BG-induced priming of macrophages. Further experiments confirmed that GM-CSF up-regulated Dectin-1 cell surface expression and amplified macrophages response along BG-mediated training. However, the blockade of GM-CSFR demonstrated that GM-CSF was not primarily required for BG-induced training of macrophages although it can substantially improve it. In addition, we found that mouse macrophages trained with BGs upregulated their expression of the four and a half LIM-only protein 2 (*Fhl2*) in a Dectin-1-dependent manner. Consistently, we observed that intracellular levels of FHL2 increased after stimulation of macrophages with BGs. In conclusion, our experiments provide new insights on GM-CSF contribution to the training of cells from the monocytic lineage and highlights FHL2 as a possible regulator of BG-associated signaling.

## Introduction

The immune system has the complex task of detecting invading pathogens, a critical step in mounting efficient mechanisms of defense. For that, innate immunity has evolved an elaborated system of pathogen surveillance with a wide variety of receptors also referred as pathogen recognition receptors (PRRs) encompassing toll-like and C-type lectin receptors (TLRs and CLRs) (1, 2). These receptors are highly expressed by innate immune cells from the monocytic lineage and are able to recognize a broad spectrum of highly conserved micro-organisms-associated molecular patterns (MAMPs). The nature of MAMPs shapes the immune response orchestrated by macrophages and dendritic cells (3). Among CLRs, Dectin-1 is essential for mounting an effective innate immune response to fungal pathogens, as demonstrated *in vivo* by several authors using *Clec7a-*deficient mice (4–6). The recognition of β-glucans (BGs) from the cell wall (CW) of various fungi, including yeasts, by Dectin-1 induces a Syk/CARD9 signaling cascade (7–11). Soluble BGs from *Grifola frondosa* have also been shown to stimulate the production of GM-CSF, a hematopoietic growth factor that could mediate part of their immunostimulant activity (12, 13). Indeed, in collaboration with Dectin-1 engagement, GM-CSF was shown to synergistically and robustly initiate a BG-specific inflammatory response in macrophages as well as in dendritic cells (12–15).

Moreover, it was demonstrated that TLR and Dectin-1-associated signaling pathways could synergize to enhance macrophages response against pathogenic fungi as *Candida albicans, Aspergillus fumigatus*, and *Pneumocystis carinii* (6, 16–19). Indeed, the combination of TLR2/4 and Dectin-1-dependent stimuli, such as those constituting yeasts CW, including mannans, phospholipomannans, and BG, triggers a strong activation of macrophages secreting high levels of inflammatory cytokines (17, 20–22). However, we recently demonstrated that preferentially targeting Dectin-1 through BG enrichment from *Saccharomyces cerevisiae* (*Sc*) CW only elicit low or no relevant cytokine or chemokine production in mouse macrophages (23). And yet, several studies have brought pieces of major evidence that pre-exposure to *C. albicans* or *C. albicans*-derived BG could enhance the response of human monocytes to a secondary stimulation with TLR ligands, while respecting a 6-day resting period between pretreatment and re-stimulation (24, 25). This effect is now referred as BG-mediated immune training of monocytes (26, 27). Recent findings highlighted some molecular mechanisms involved in this long-term trained immunity model, including a metabolic shift toward aerobic glycolysis (a feature of cell activation and proliferation) *via* PI3K/AKT/mTOR pathway (28) and epigenetic modifications (29) in BG-trained human monocytes. Although GM-CSF priming was very recently shown to increase responsiveness to lipopolysaccharide (LPS) in a short-term model of training (30), less is known regarding its role in BG-related trained immunity.

If producible to a large scale, *C. albicans* BG could be highly promising molecules to develop immunotherapeutic strategies where reprogramming of monocytes is required. Thus, we thought that *Sc* BG, presenting a quite analogous structure to those from *C. albicans* and industrially obtainable, could be used as surrogates, provided they share a similar ability to prime mononuclear phagocytes. In the perspective of deciphering the mechanisms underlying this effect and more convenient *in vivo* investigations, we attempted to establish a short-term model of trained immunity (devoid of resting period between both stimulations) with mouse macrophages.

By combining cellular and molecular approaches, we confirmed that pre-exposure of mouse macrophages with *Sc* BG promoted intense cytokine production upon secondary stimulation with TLR agonists. Through microarray analysis, we highlighted significant transcriptional modifications specific from BG pretreatment. Among these, *Csf2* and *Fhl2* over-expression was further investigated to evaluate their potential contribution to BG-induced priming of mouse macrophages.

## Materials and Methods

### Reagents

Cell culture media RPMI 1640 GlutaMAX™ and DMEM GlutaMAX™, PBS, non-essential amino acids (NEAA), sodium pyruvate and antibiotics [Penicillin–Streptomycin (Pen–Strep™), Gentamicin™, Normocin™, and Zeocin™] were purchased from GIBCO (Life Technologies). Fetal bovine serum (FBS) was provided by Eurobio, France. Zymosan (Zym), particulate and dispersible whole glucan particle (WGPd, Biothera) and soluble whole glucan particle compounds (WGPs, Biothera) and curdlan (Curd), a linear β1,3-glucan extracted from *Alcaligenes faecalis*, synthetic triacylated lipoprotein Pam3CSK4 (Pam3) and ultraPure LPS from *Escherichia coli* O111:B4 were purchased from InvivoGen (France). BG compounds of interest were extracted from the same strain of *S. cerevisiae* owned by Phileo-Lesaffre Animal Care. Their composition was already described in a recent study (23). The previous name given to the crude compounds BG15 was replaced in this study by ScCW for better understanding and more convenience.

### Animals

Wild type (WT) C57Bl/6 mice were purchased from Janvier Labs (St Berthevin, France) and *Clec7a*^−/−^ mice (5) were originally provided by Pr. Gordon Brown (University of Aberdeen, Scotland) and were bred in-house. Eight- to 12-week-old C57Bl/6 *Clec7a*^−/−^ mice and their strain-matched WT controls from both sexes were housed under pathogen-free conditions in an accredited research animal facility of the National Veterinary College (UMR IHAP, Toulouse, France). This study was carried out in strict accordance with the Federation of European Laboratory Animal Science Association guidelines (FELASA). Experiments were performed by FELASA accredited investigators (no. 311155580) and approved by the local ethics committee, “Science et Santé Animale” (SSA). All efforts were made to minimize animal pain and distress.

### Cells and Bacteria

#### NFκB/AP-1 Activity Using Reporter Cell Line

The NFκB/AP-1 reporter RAW-Blue™ Cells (InvivoGen™) were cultured and propagated according to the manufacturer’s recommendations. The NFκB/AP-1 activity was assessed as described in *Walachowski* et al. (23). RAW-Blue™ macrophages (1 × 10^5^ cells/well) were stimulated with 100 µg/mL of BG-containing preparations (ScCW, BG65, and BG75) for 8 h. Supernatants were then removed and 100 ng/mL of ultraPure LPS was added in each well for the rest of the stimulation period. Supernatants were collected and stored at −20°C or processed immediately. Secreted embryonic alkaline phosphatase was measured using a colorimetric enzymatic assay. Supernatants were incubated with Quanti-Blue™ (InvivoGen) 25% v/v. for 2 h at 37°C, and optical density (OD 650 nm) was measured (VERSAmax plate reader, Molecular Devices).

#### Bacteria

*Staphylococcus aureus* N305 and *E. coli* P4 strains were prepared as described previously by Accarias et al. (31) using growth medium adapted to each strain of bacteria [tryptic soy broth for N305 and Lysogeny broth (LB) for P4]. Briefly, a 100-fold dilution of the overnight bacteria culture was grown in medium to mid log phase to obtain an OD600 nm of around 1. After cautious washing and homogenization, the concentration of bacteria was estimated by measuring the absorbance at 600 nm (considering that 1 D.O. unit corresponds to 5 × 10^8^ CFU/mL) and was adjusted to the desired concentration. CFUs were further determined in serially diluted inoculum after 24 h of culture on agar plates.

#### Primary Cell Culture and Functional Assays

Murine wild type or *Clec7a*^−/−^ bone marrow-derived macrophages (BMDM) were obtained as described previously (31). Inflammatory peritoneal macrophages were elicited and handled as previously described (23). BMDM and thioglycollate-elicited peritoneal macrophages (TEPM) (1 × 10^5^ cells/well) were seeded in 96-well tissue culture plates for 16 h in complete RPMI until complete adherence (37°C, 5% CO_2_). Non-adherent contaminating peritoneal cells were eliminated by repeating three gentle washings of wells with pre-warmed culture medium or PBS. After stimulation with *Sc* BG compounds or Dectin-1 ligands controls (WGPd, WGPs or Zym) for 8 h, supernatants were removed and cells were then stimulated with 100 ng/mL of ultraPure LPS or Pam3, or with live bacteria (*S. aureus* N305 or *E. coli* P4 strains, MOI = 10) for 1 h followed by 16 h incubation with cell culture medium supplemented with Gentamicin™. Supernatants were then collected, complete protease inhibitor cocktail (Roche, France) was added in infected conditions, and supernatants were finally stored at −20°C or −80°C until further use.

For the dose-dependent experiments, BMDM were pre-incubated with 1:10 serial dilution of BG75 from 0.1 to 100 µg/mL before another 16 h LPS stimulation. For the two kinds of kinetics assays, several time points were used for BG incubation (1, 4, 8, or 16 h) before 16 h of LPS stimulation as well as for LPS stimulation (1, 4, 8, or 16 h) after 8 h of BG incubation.

For short-term model of immune training, cells were pretreated with BG75 or complete RPMI as above. After the first incubation, BMDM were washed with warm PBS and maintained in complete RPMI for 24 or 72 h. Thereafter, cells were submitted to a secondary stimulus using 100 ng/mL of ultraPure LPS for 16 h. For GM-CSF and M-CSF influence analyses, BMDM were first pre-incubated for 2 h with recombinant GM-CSF (rGM-CSF, 5 ng/mL, PeproTech, Rocky Hill, NJ, USA) or M-CSF (rM-CSF, 5 ng/mL, PeproTech, Rocky Hill, NJ, USA), or using serial 10-fold dilutions as described in corresponding figure legends. Cells were then stimulated with 100 or 10 µg/mL of BG75 for 8 h followed, where applicable, by 16-h LPS stimulation.

For blocking antibody assays, macrophages were pre-incubated with blocking anti-GM-CSFR antibodies or their isotype control (Novus Biologicals, CO, USA) for 1 h, followed by incubation with rGM-CSF (100 pg/mL) for 2 h. Then BMDM were treated with BG75 (100 µg/mL) for 8 h and further stimulated with 100 ng/mL of LPS for 16 h. All triplicate supernatants were harvested and handled as previously described before cytokine measurement.

### Quantification of Cytokines and Chemokines by ELISA

TNFα, IL-6, and IL-1β (Biolegend, Ozyme-France) and GM-CSF (R&D Systems, France) levels in culture supernatants were assayed using individual cytokine detection kits according to the manufacturer’s recommendations. Data are expressed as the mean ± SD and are representative of three individual experiments performed in triplicate.

### Dectin-1 and CD11b Surface Expression Analysis by Flow Cytometry

Wild-type BMDM were incubated with rGM-CSF (5 ng/mL) or rM-CSF (5 ng/mL) or medium for 2 h. Supernatants were discarded and cells were collected using cold PBS supplemented with 5 mM EDTA. After harvest, cells were centrifuged (300 g, 5 min) and absolute macrophages number was determined by flow cytometry (MACSQuant, Miltenyi Biotech, Germany). BMDM were pre-incubated with anti-CD16/CD32 (Biolegend, Ozyme-France) to block FcγRII/III receptors and then incubated with the following fluorochrome-conjugated mAbs: anti-Dectin-1 (2A11; AbD serotec) or its isotype control IgG1 (A110-1, BD biosciences Pharmingen) and anti-CD11b (M1/70, Biolegend) or its isotype control. A 7-AAD staining (Biolegend, Ozyme-France) was used to discriminate death cells and doublets of cells were excluded with a gating on FSC-H/FSC-A. The acquisition was performed on 1 × 10^5^ cells using MACSQuantify software (Miltenyi Biotech, Germany). Data were analyzed with FlowJo software (FlowJo LLC, USA).

### Microarray Analysis

After stimulation with *Sc* BG compounds and then with ultraPure LPS, BMDM were lysed in Buffer RLT (Qiagen, Hilden, Germany) and was subjected to RNA extraction using the RNeasy Mini Kit (Qiagen, Hilden, Germany) according to the manufacturer’s instructions. RNA was quantified with a NanoDrop^®^1000 spectrophotometer and NanoDrop 1000.3.7 software.

RNA quality assessment and microarray experiment were performed at the GeT-TRiX platform (INRA, Toulouse, France). RNA quality was assessed using the Agilent RNA 6000 Nano Kit on BioAnalyzer and 2100 Expert Software (Agilent Technologies, Santa Clara, CA, USA). RNA samples with RNA integrity number more than 8.5 were chosen to be prepped for further analysis. A total RNA material of 100 ng was amplified and labeled using a Low Input QuickAmp Labeling kit (Agilent Technologies, Santa Clara, CA, USA). RNA were hybridized to Agilent Sure Print G3 Mouse GE 8 × 60K microarrays, washed, stained, and scanned on an Agilent G2505C instrument following the manufacturers’ protocols. Agilent Feature Extraction software was used to analyze signal intensity values of the spots generated from the scans. Post-hybridization quality controls were done to eliminate outliers and irrelevant data from the expression data set. Background was subtracted and data were normalized using Agilent procedure. All validated and normalized transcripts expression data were processed using the Agilent GeneSpring GX software.

All entities with flag values present in at least 1 out of the eight conditions were considered. Statistical analysis (two-way ANOVA) was used to generate a unique list of up- or downregulated entities with associated Benjamini–Hochberg false discovery rate corrected *p*-value and fold change (FC) and for hierarchical clustering. Transcripts with detection *p*-value of less than or equal to 0.001 in at least one sample were selected for further analysis. A filter was set to include only transcripts that had at least 1.5-FC compared to the LPS-stimulated cells without BG treatment control.

Using Ingenuity Pathway Analysis (IPA) software, a final list of unique identified genes (*p*-value <0.001 and absolute FC ≥2) was generated after selection of mapped entities and deduplication exercise on them, and which was then used to perform IPA bio-function analysis. IPA categorized modulated genes according to *p*-values (calculated by the Fisher exact test) and *z*-scores. The *z*-score predicts the direction of change of a function: a function is increased when *z*-score is ≥2 and decreased when *z*-score ≤2. IPA also calculated a bias-corrected *z*-score to correct dataset bias (i.e., when there are more up- than downregulated genes in a bio-function or vice-versa). Lists of modulated genes (FC ≥1.5) were also processed using InnateDB online tool to identify the biological functions associated with the primary BG compounds treatment (*p*-value <0.001). All data files have been deposited in NCBI’s Gene Expression Omnibus and are accessible through GEO series accession number GSE101959 (https://www.ncbi.nlm.nih.gov/geo/query/acc.cgi?acc=GSE101959).

### Gene Expression by Quantitative Polymerase Chain Reaction Analysis

Total RNA (300 ng) was reverse transcribed using the SuperScript III First-Strand Synthesis Super Mix Kit (Invitrogen) as per the manufacturer’s protocol. Quantitative PCR was performed individually with Power SYBR Green PCR Master Mix (Applied Biosystems) and LightCycler™ 480 (Roche, France) for *Csf2* and *Fhl2*; or with the Biomark HD System (Fluidigm, France) at the GeT-PlaGe genotyping service platform (INRA, Toulouse, France) according to the manufacturer’s recommendations. For individual quantification, dissociation curves analysis was performed at 40 cycles to verify the identity of PCR product. Primer3plus software was used to design the primers (Table S1 in Supplementary Material) and housekeeping genes were selected with GeNorm Software. The abundance of mRNA of interest was normalized to that of *Sdha, Rpl9*, and *Hprt1* and relative expression was calculated using the 2^−ΔΔCt^ method. The comparative threshold cycle values are expressed as arbitrary unit.

### Western Blot

After 8 h of *Sc* CW extracts stimulation, WT BMDM were exposed to 100 ng/mL of ultraPure LPS for 16 h. Supernatants were removed and cells were then lysed in RIPA lysis buffer supplemented with 5 mM EDTA and protease inhibitor (Pierce™, USA). Lysates were centrifuged (14000 rpm, 10 min), pellets were discarded and proteins concentrations were determined using BCA kit (Pierce™, USA). Following a 1:1 dilution in Laemmli buffer (Biorad, CA, USA), 10 µg of total protein were separated on NuPAGE^®^ 4–12% Bis-Tris Mini gels (1.0 mm, 12 wells, Invitrogen™) and blotted onto nitrocellulose membrane (0.2 µm, Invitrogen™) using the XCell *Sure*Lock™ Mini gel system (Invitrogen™, USA). After blocking with 1% BSA and 0.05% Tween 20 (Sigma-Aldrich^®^, USA) in Tris-Buffer Saline (TBS, Euromedex, France), the membrane was stained with primary anti-FHL2 (F4B2-B11) and anti-β-actin (C4) as loading control mAbs (Santa Cruz Biotechnologies, Germany) at 4°C, overnight. The membrane was then washed in TBS with 0.05% Tween 20 and incubated with goat anti-mouse antibodies conjugated with horseradish peroxidase (Jackson ImmunoResearch, PA, USA) for 1 h at room temperature. Signals were detected with Clarity™ Western ECL substrate and the ChemiDoc™ MP instrument according to the manufacturer’s instructions (Biorad, CA, USA). Image Lab software was used for minor linear adjustments in contrast, if needed and the quantitative tool was used to determine the relative quantity of FHL2 protein in each sample as compared to the reference value (fixed to 1) corresponding to the untouched macrophages condition.

### Statistical Analysis

All experiments were performed three times unless otherwise specified and data are expressed as the mean ± SD of the values from all experiments. Each condition was performed in triplicate. Statistical significance was assessed using a two-tailed unpaired Student’s *t*-test or a two-way ANOVA analysis (as stated in the figure legends) with a threshold set at *p* < 0.05. Mean values shown with different letters on plots are significantly different. For these analyses, we used XLStat (Addinsoft, France) and GraphPad Prism 5 (San Diego, CA, USA) softwares.

## Results

### Priming with *S. cerevisiae* BGs Enhances the Macrophage Response to Secondary Stimulation with TLR Ligands in a Dectin-1-Dependent Manner

**Figure 1:**
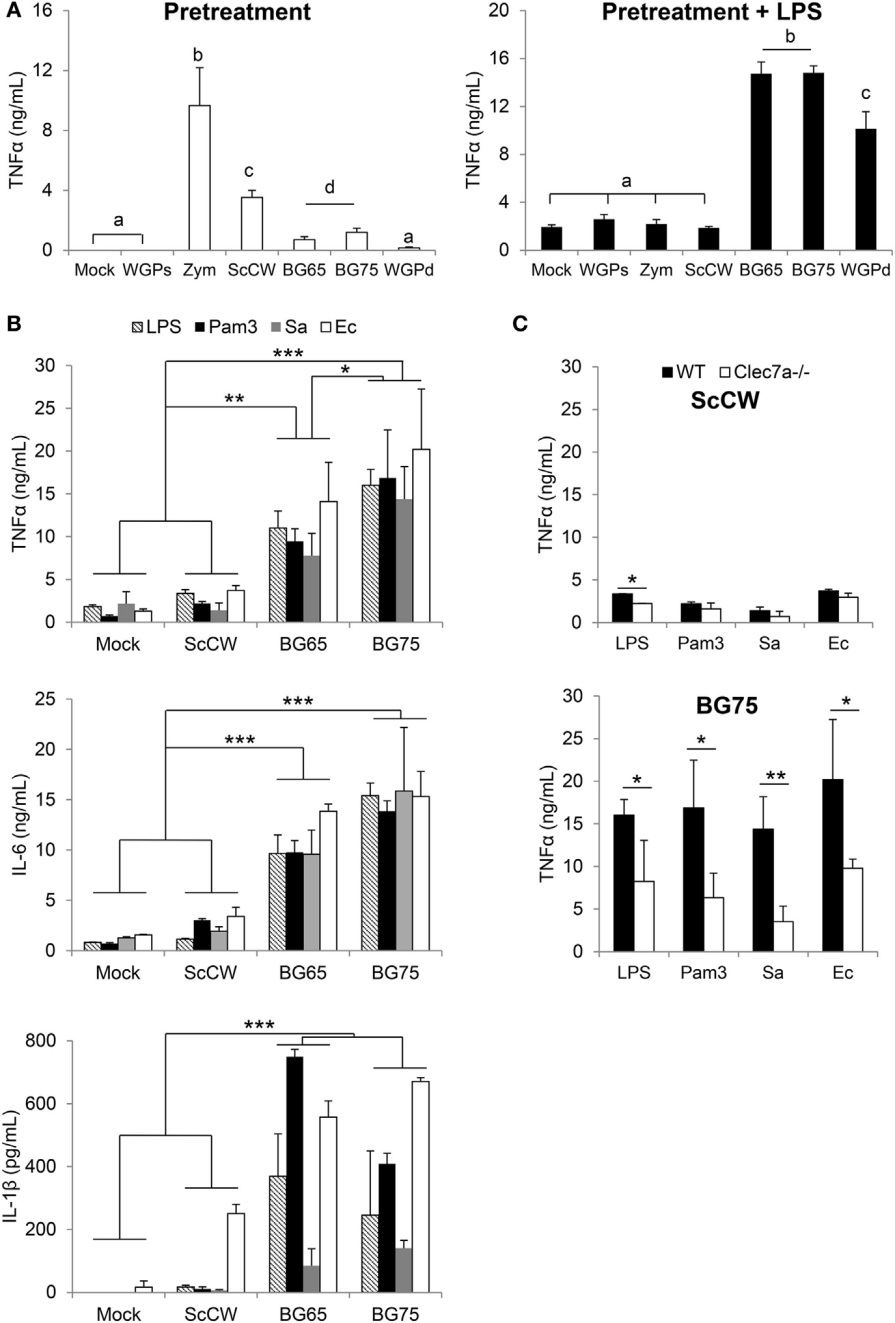
BG-enriched preparations of *Saccharomyces cerevisiae* cell wall (ScCW) prime the macrophage response predominantly *via* Dectin-1 upon toll-like lectin receptor (TLR) ligands or whole bacteria exposure. Wild-type (WT) thioglycollate-elicited peritoneal macrophages **(A)** and WT or *Clec7a*^−^*^/^*^−^ bone marrow-derived macrophages from C57BL/6 mice **(B,C)** were stimulated with 100 µg/mL of crude (ScCW) or BG-enriched (BG65, BG75) compounds for 8 h. After incubation, cell culture supernatants were collected [panel **(A)** on the left, white bars] and stored at −20°C. Cells were then stimulated with 100 ng/mL of ultraPure lipopolysaccharide (LPS) for 16 h [panel **(A)** on the right: dark bars, **(B)** and **(C)**] or Pam3CSK4 (Pam3) [**(B)** and **(C)**]. For the stimulation with live bacteria, *Staphylococcus aureus* N305 or *Escherichia coli* P4 strains were used at MOI = 10 for 1 h, followed by 16 h incubation with cell culture medium supplemented with Gentamicin™ **(B)** and **(C)**. At the end of the incubation, supernatants were collected and immediately stored at −20°C. TNFα, IL-6, and IL1β were quantified by ELISA. Data are expressed as the mean ± SD from three independent experiments performed in triplicate. In **(A,C)**, a Student’s *t*-test was used to assess significance between conditions (*p < 0.05; **p < 0.01) and mean values not sharing the same letter in **(A)** are significantly different. In **(B)**, a two-way ANOVA with Bonferroni post-tests was used to compare pretreatment conditions (*p < 0.05; **p < 0.01, ***p < 0.001).

*In vitro*, pre-incubation of TEPM with various yeast CW-derived products resulted in different degrees of cytokine production. As previously published (23), unpurified or poorly purified products such as Zym or crude CW induced strong cytokine secretion as measured in the culture supernatants (Figure 1A). In comparison, the secretion induced by particulate BG-enriched products was lower but still significant if compared to mock or soluble BG (WGPs) which seemed devoid of activity. The results appeared strikingly different when pre-incubated macrophages were further stimulated with LPS. Indeed, pre-treating TEPM with BG (BG65, BG75, and WGPd) enhanced their response to LPS by at least a factor of 10 compared to untreated cells or cells pretreated with WGPs. Surprisingly, ScCW or Zym preparations, despite the presence of low concentrations of BG, did not amplify TEPM response to LPS. We next confirmed these observations by using BMDM pre-incubated with crude CW (ScCW) or purified BG (BG65/BG75) and further stimulated with various TLR agonists (Figure 1B). As with TEPM, pretreating BMDM with BG dramatically enhanced their response to TLR agonists whether these were presented to macrophages as purified molecules (TLR4: LPS, TLR2: Pam3) or in the context of living Gram-positive or Gram-negative bacteria (*S. aureus* and *E. Coli*, respectively). This effect was observed for the three inflammatory cytokines TNFα, IL-6, or IL-1β, and again, pretreatment with ScCW (or Zym, data not shown) did not allow significant priming of the macrophage response. Given significant levels of IL-1β measured in BG-enriched pretreatment conditions, we evaluated the viability of BMDM using Propidium Iodide labeling assay. Any difference of fluorescence between mock and BG75-pretreated experimental conditions was detected before or after LPS stimulation, meaning that cells were perfectly viable in our short-term training model (Figure S1 in Supplementary Material).

**Figure 2:**
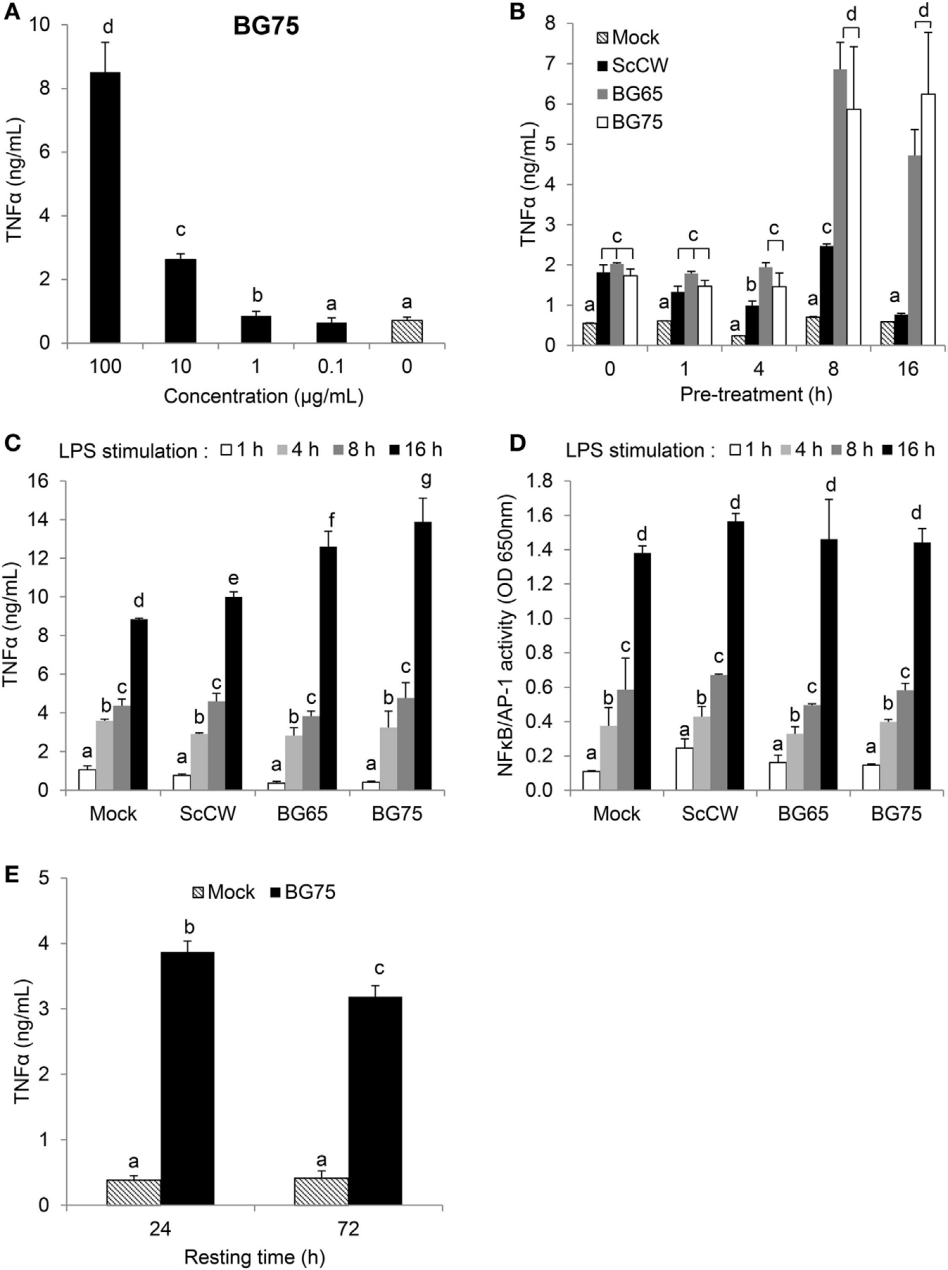
β-glucans (BGs) prime the production of TNFα in macrophages *via* a late and dose-dependent mechanism lasting beyond BG incubation. **(A,B,E)** bone marrow-derived macrophages (BMDM) were subcultured in 96-well plates. **(A)** Cells were incubated for 8 h with a 10-fold serial dilution from 100 to 0.1 µg/mL of BG75. After supernatant removal, BMDM were then stimulated with 100 ng/mL of ultraPure lipopolysaccharide (LPS) for 16 h. Supernatants were collected at the end of the incubation for further analysis. **(B)** Cells were stimulated for 1, 4, 8, 16, and 24 h with 100 µg/mL of BG-containing products (ScCW, BG65, and BG75). Supernatants were discarded and 100 ng/mL of ultraPure LPS was added in each well for 16 h incubation. Supernatants were immediately collected and stored at −20°C. TNFα was measured using ELISA and data are expressed as the mean ± SD from two independent experiments performed in triplicate. **(C,D)** NFκB/AP-1 reporter RAW-Blue™ macrophages were treated with 100 µg/mL of BG-containing compounds for 8 h before stimulation with 100 ng/mL of ultraPure LPS for 1, 4, 8, or 16 h. At the end of the incubation, supernatants were harvested and stored at −20°C until further use. NFκB/AP-1 activity was determined by a colorimetric enzyme assay where cell culture supernatants were incubated with Quanti-Blue™ reagent before reading OD at 650 nm. TNFα was quantified by ELISA. Data are expressed as the mean ± SD from three independent experiments performed in triplicate. **(E)** BMDM were incubated for 8 h with 100 µg/mL of BG75 or control. After supernatant removal, fresh medium was added for further 24 or 72 h (resting time) and cells were stimulated with 100 ng/mL of ultraPure LPS for 16 h. Supernatants were collected and stored at −20°C. TNFα was measured using ELISA and data are expressed as the mean ± SD from three independent experiments performed in triplicate. Mean values not sharing the same letter are significantly different according to the Student’s *t*-test (*p* < 0.05). For **(B–D)**, results of statistical analyses are displayed for comparisons between each compound according to the incubation time.

Bone marrow-derived macrophages from *Clec7a*^−/−^ mice, which do not express Dectin-1, the main surface receptor of BG, were then used to evaluate the influence of the Dectin-1 pathway in the ability of BG to prime macrophages. Whereas the absence of Dectin-1 had no effect on the ScCW-mediated response, it significantly inhibited the ability of BG to prime BMDM response against TLR agonists (Figure 1C). Nevertheless, this inhibition was only partial and led to a reduction by 60% on average of cytokine production. Moreover, the surface expression of TLR4 on BMDM was evaluated after BG pretreatment, and we showed that the BG-induced exacerbated cytokine response observed following a secondary LPS stimulation is not explained by an upregulation of TLR4 levels at the surface of BMDM (Figure S2 in Supplementary Material). Of interest, priming of macrophages was perfectly correlated with the dose of BG used during pretreatment (Figure 2A). We determined that 1 µg/mL was enough to prime macrophages although concentrations higher than 10 µg/mL were significantly more efficient. Next, we evaluated the incubation time needed for BG to exert their priming effect on the macrophage response to secondary stimuli. First, the simultaneous addition of BG or ScCW and LPS (co-stimulation) or short pre-incubation times (up to 4 h) only resulted in a slight increase of the macrophage response reflecting the previously described synergism between TLR and Dectin-1 pathways (6, 17, 19). By contrast, longer pre-incubation times allowed a significant priming of macrophages but exclusively when using BG-enriched preparations (Figure 2B). In parallel, we also assessed importance of the time for the secondary stimulation to maximize cytokine production. By doing so, we observed that, as expected, TNFα levels produced in response to LPS stimulation rose with the incubation time (Figure 2C). However, for shorter LPS incubation times (from 1 up to 8 h), no difference was noticed between TNFα levels produced by untreated or BG-pretreated macrophages. Thus, at least 16 h of incubation with LPS were needed to observe significant differences between pretreatment conditions. A good correlation was identified between NFκB/AP-1 activity and incubation times after LPS stimulation. However, after 16 h of incubation, the levels of TNFα secretion and NFκB/AP-1 activity did not match (Figure 2D), suggesting that the increased secretion of cytokine induced by BG pretreatment did not rely on NFκB/AP-1 activity. Finally, we evaluated how long the priming effect of BG on macrophages lasted. As shown in Figure 2E, enhancement of the macrophages response to LPS could be observed at least up to 72 h after BG pretreatment. This was also observed for longer latency times and up to 5 days (data not shown). Taken together, these results indicate that BG priming is a slow process that probably involves *de novo* protein synthesis and induces profound and long-lasting changes in macrophage biology and phenotype.

### Transcriptome Analysis Reveals Specific Hallmarks of BG-Primed Macrophages

**Figure 3:**
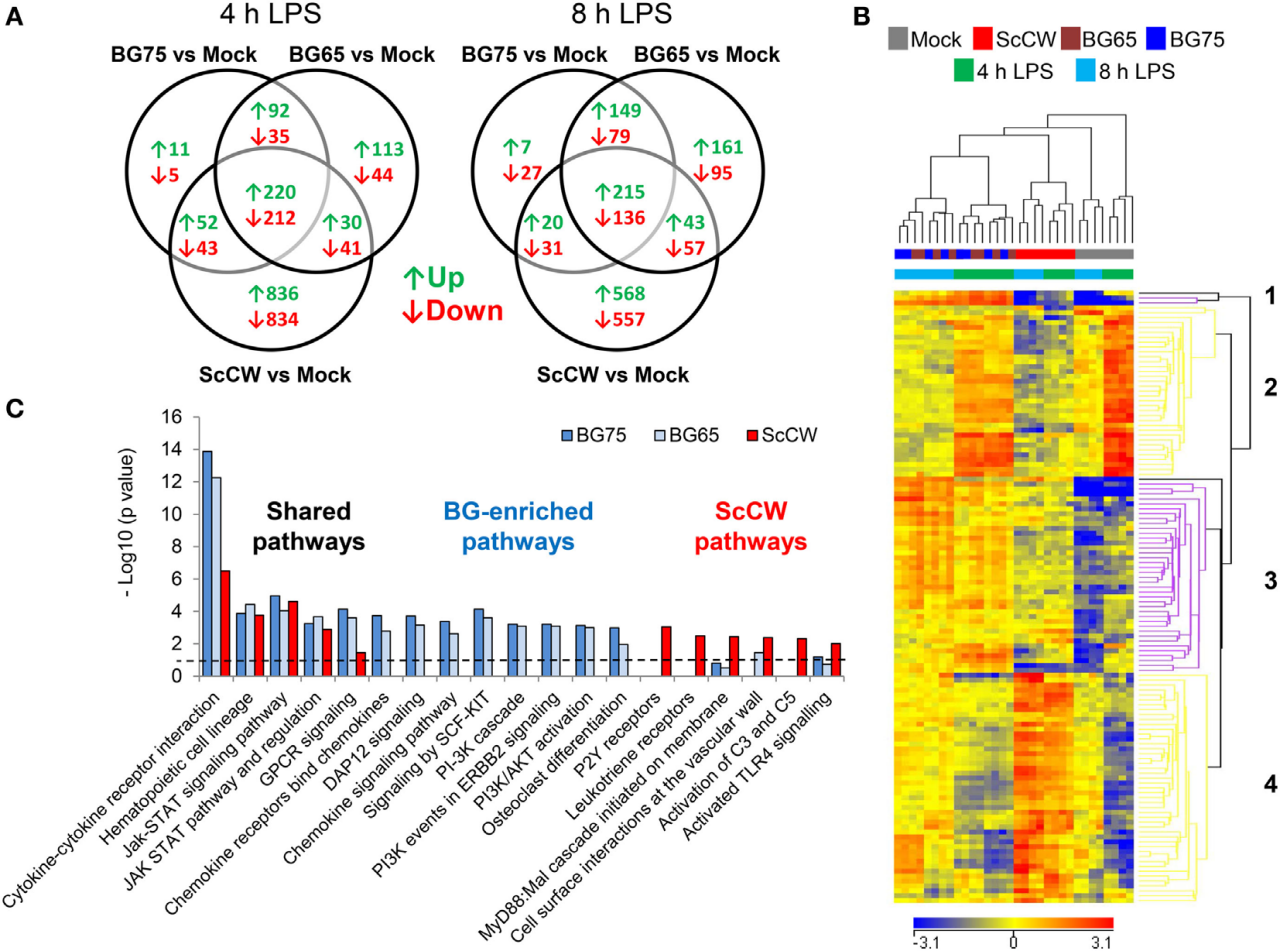
Gene expression profiling reveals specific hallmarks of β-glucan (BG)-pretreated macrophages after lipopolysaccharide (LPS) exposure. Wild-type bone marrow-derived macrophages (BMDM) were cultured in 24-well plates and treated with 100 µg/mL of the set of three BG extracts for 8 h in triplicate. After incubation, supernatants were removed and cells were stimulated with 100 ng/mL of ultraPure LPS for 4 or 8 h. A microarray analysis was performed using Agilent mRNA transcriptomic analysis. After quality control and normalization, data were analyzed using Agilent GeneSpring GX software by two-way ANOVA and Benjamini–Hochberg *post hoc* correction. Transcripts with a *p*-value of less than or equal to 0.001 and a fold change (FC) value of at least 1.5 between conditions in at least one sample were selected for further analysis. **(A)** Venn diagrams showing the overlap of upregulated (↑) and downregulated (↓) genes in each condition were obtained using VENNY 2.0. **(B)** Unsupervised hierarchical clustering (Euclidian distance) of transcriptional profiles, displayed as a heatmap of FC expression values, from BG-treated BMDM compared to non-treated BMDM upon LPS exposure. Each row represents a transcript and each column an individual sample. The heatmap shows 4 clusters constituted by entities with a *p*-value of 0.001 for which FC were greater than 5 compared to mock plus 8 h LPS-stimulated cells. Red indicates over-expressed and blue indicated under-expressed transcripts. **(C)** Based on a comparison of FC expression values between BG pretreatments and the non-pretreated condition upon 8 h of LPS exposure obtained from GeneSpring software (FC >1.5), we performed a pathway over-representation analysis using Innate DB. Significant pathways which *p*-values (calculated by the hypergeometric algorithm) were lower than 0.05 are displayed for each BG pretreatment condition (threshold value–log *p*-value = 1.30).

To get further insight on how BG altered macrophages responsiveness, we performed a gene expression analysis by microarray. Gene expression profiling was performed in BMDM pretreated with ScCW, BG65, and BG75, or mock controls for 8 h, and then stimulated with LPS for 4 and 8 h. Using a two-way ANOVA with a false discovery rate of 0.001 and a fold expression difference of at least 1.5, we determined differentially expressed genes (DEG) between conditions of priming: BG65, BG75, or ScCW versus mock (Figure 3A). A total of 787/670 (4 h) and 935/664 (8 h) DEG were found when comparing BG65/BG75 versus mock. Comparatively, ScCW pretreatment modified the expression of a greater number of genes (2,268 and 1,627 at 4 and 8 h, respectively). Microarray results were confirmed using RT-qPCR on a different set of samples. qPCR results were strongly correlated with the microarray results for all the genes that were evaluated (*n* = 26 genes), with a Pearson’s correlation coefficient of 0.57 (*p*-value <0.001), and confirmed most of the microarray results (Figure S3A in Supplementary Material).

Based on a fold difference above 5, DEG were organized by hierarchical clustering (Euclidian distance) according to similarities in the expression profiles. Conditions with BG65 and BG75 clustered together in contrast to ScCW and mock conditions that remained separated, and so whatever the time point analyzed (Figure 3B). The discrimination was also confirmed by principal component analysis (data not shown). Two gene clusters (1 and 3) were associated with pretreatment of macrophages with BG. Cluster 1 was composed of only three differentially up-regulated genes by BG65/75: *Csf2, Fhl2, and Cish* that presented the highest FC compared to mock (Table 1, A). Interestingly, these genes were poorly modulated in ScCW pretreatment condition. Consequently, genes from cluster 1 could be considered as hallmarks of BG-enriched pretreatment of macrophages. Cluster 3 was also associated with BG pretreatment and comprised 40 DEG. However, these genes were also modified in ScCW condition but with a lower FC compared to BG pretreatment. The comparison between BG75 and ScCW pretreatments confirmed that, although ScCW contains very limited BG (15%), stimulation with BG-enriched preparations triggered specific pathways in macrophages (Table 1, B). Clusters 2 and 4 comprised genes specifically modulated by ScCW pretreatment. Cluster 2 comprised 35 genes that were downregulated in ScCW condition but precociously (after 4 h of LPS stimulation) over-expressed in mock and BG conditions. Cluster 4 comprised 47 genes significantly upregulated after 8 h of LPS exposure (and to a lesser extent at 4 h) but exclusively in ScCW-pretreated macrophages. By contrast, these genes were down-modulated in mock or BG pretreatment conditions (Table S2 in Supplementary Material). These results demonstrate that pretreatment of macrophages with BG-enriched preparations induced the modulation of a specific array of genes that were distinct from those modulated by ScCW pretreatment.

**Table 1:** Top-10 list of the most differentially expressed genes after BG75 treatment upon lipopolysaccharide (LPS) exposure.

(A) ↑Up-regulated, Pretreated versus mock (fold change)			4 h LPS			8 h LPS		
Rank	Gene	Gene title	*Saccharomyces cerevisiae* cell wall (ScCW)	BG65	BG75	ScCW	BG65	BG75
1	*Csf2*	Colony stimulating factor 2 (granulocyte-macrophage)	1.8	20.3	14.0	4.2	64.9	43.1
2	*Fhl2*	Four and a half LIM domains 2	2.4	43.3	31.1	1.7	41.0	35.7
3	*Hbegf*	Heparin-binding EGF-like growth factor	2.8	11.3	8.3	6.0	46.0	32.6
4	*Inhba*	Inhibin beta-A	16.3	20.1	15.3	20.9	41.0	29.9
5	*Car2*	Carbonic anhydrase II	10.9	29.2	19.6	9.8	36.9	27.2
6	*Dok7*	Docking protein 7	9.4	19.6	13.1	11.8	34.0	22.9
7	*Csf3*	Colony stimulating factor 3 (granulocyte)	32.1	11.4	12.7	15.2	21.2	19.2
8	*Cish*	Cytokine inducible SH2-containing protein	-2.9	3.0	2.1	1.5	19.8	13.0
9	*F3*	Coagulation factor III	1.8	5.6	4.8	2.1	13.1	11.4
10	*Vasn*	Vasorin	1.6	4.5	3.3	3.2	13.3	10.8

**(B) ↑Up-regulated, β-glucan (BG)- versus *Saccharomyces cerevisiae* cell wall-pretreated (fold change)**				**4 h LPS**			**8 h LPS**	
**Rank**	**Gene**	**Gene title**		**BG65**	**BG75**		**BG65**	**BG75**

1	*Fhl2*	Four and a half LIM domains 2		18.0	13.0		24.1	20.9
2	*Csf2*	Colony stimulating factor 2 (granulocyte-macrophage)		11.3	7.8		15.5	10.3
3	*Cish*	Cytokine inducible SH2-containing protein		8.7	5.9		13.1	8.6
4	*Lrrc32*	Leucine rich repeat containing 32		8.2	6.0		11.0	8.0
5	*Ccl17*	C-C motif chemokine ligand 17		6.4	4.3		10.3	6.6
6	*F3*	Coagulation factor III (thromboplastin, tissue factor)		3.2	2.7		6.4	5.5
7	*Hbegf*	Heparin-binding EGF-like growth factor		4.0	2.9		7.6	5.4
8	*Tnfsf18*	Tumor necrosis factor superfamily member 18		1.7	1.8		4.9	4.8
9	*Spry1*	Sprouty RTK signaling antagonist 1		1.8	1.5		5.1	4.7
10	*Egr2*	Early growth response 2		4.3	3.4		5.4	4.7

Based on these panels of DEG, we performed a functional analysis using InnateDB online tool to uncover macrophage pathways that were significantly influenced by each pretreatment. Among the pathways identified (with a corrected *p*-value <0.05), some were clearly specific to each pretreatment whereas some were shared (Figure 3C; Figure S3B in Supplementary Material). JAK–STAT signaling and regulation and hematopoietic cell lineage pathways were targeted by BG and ScCW pretreatments in a similar extent. By contrast, although commonly targeted, cytokine–cytokine receptor interactions and G-protein-coupled receptors (GPCRs) signaling (strongly associated with *Csf2* overexpression) appeared much more induced by BG65/75 pretreatments and this was compatible with the enhanced cytokine response observed (Figure 1). Compared to BG-enriched conditions, ScCW showed a significant enrichment for TLR signaling pathways associated with MyD88 cascade, complement activation, and also functions linked to leukotriene and P2Y (Purinergic GPCRs) receptors (GPCRs signaling). This result confirmed that ScCW pretreatment significantly stimulated TLR cascade in macrophages (Figure 1A). By contrast, we identified functions specifically triggered by BG pretreatment mainly related to PI3K/AKT pathway (cascade, activation, and events in ERBB2, SCF-KIT, and DAP12 signaling). In line with this result, we observed a significant increase of NAD^+^/NADH ratio in macrophages following BG75 exposure, a biological feature linked to aerobic glycolysis (Warburg effect) and activation of cell metabolism through PI3K/AKT signaling (Figure S4 in Supplementary Material). Moreover, several genes coding for chemokines and chemokines receptors (*Cxcl1, Cxcl2, Cxcl5, Ccl17*, or *Cxcr6*, etc.) were upregulated by BG pretreatment leading to enrichment of this pathway in our analysis. Finally, osteoclasts differentiation pathway appeared significantly and specifically associated with BG condition as a result of the overexpression of *Fhl2*.

### GM-CSF Enhances BG Signaling but Is Not Primarily Involved in the Improvement of BG-Primed Macrophage Response to a Secondary TLR Stimulus

**Figure 4:**
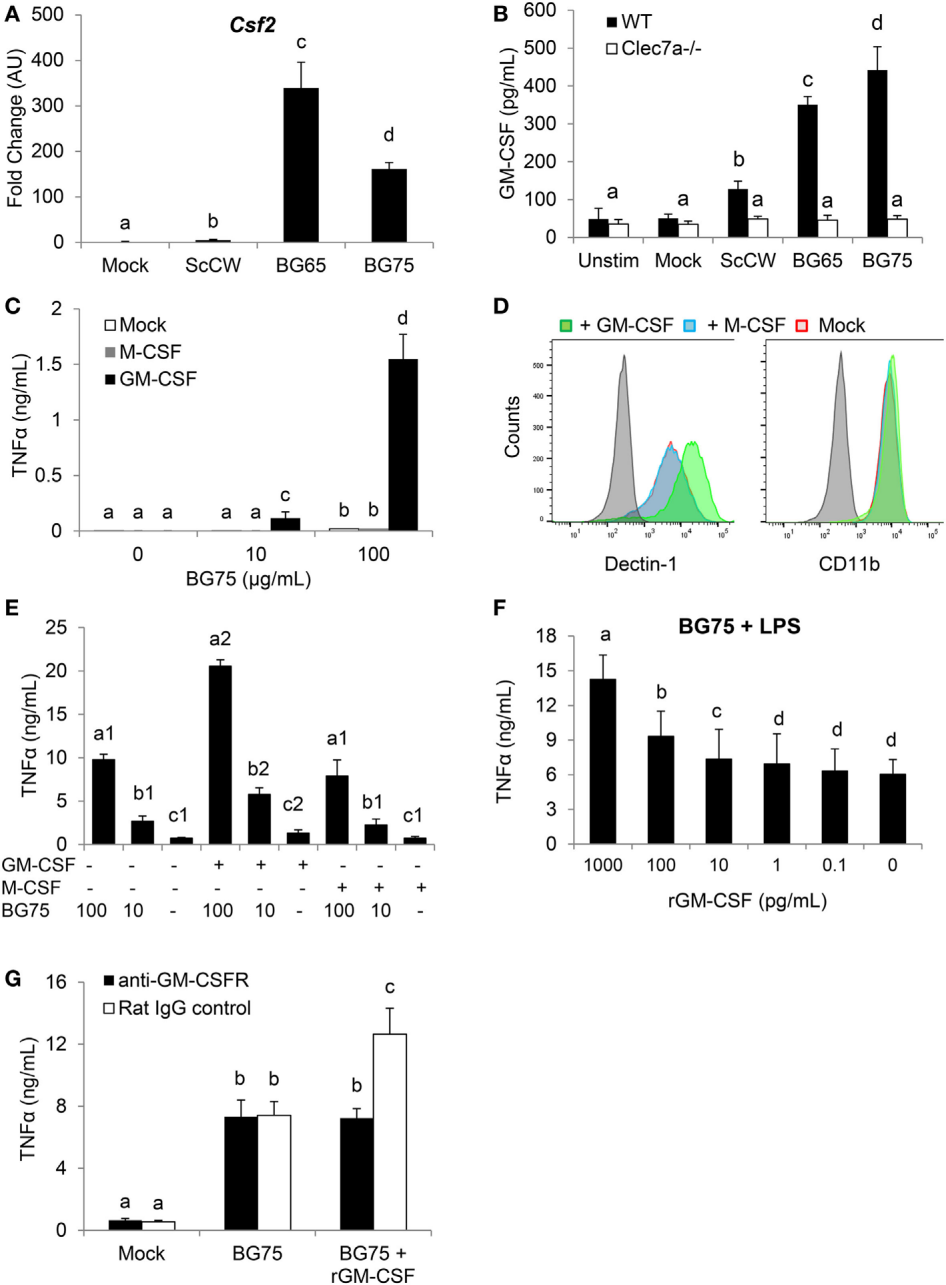
*Csf2* upregulation induced by BG-enriched compounds treatment and lipopolysaccharide (LPS) exposure is mainly Dectin-1-dependent. Wild-type or *Clec7a*^−/−^ bone marrow-derived macrophages (BMDM) were stimulated with 100 µg/mL of the various BG-containing compounds for 8 h. After incubation, cell culture supernatants were removed and 100 ng/mL of ultraPure LPS or medium was added in each well for 8 h **(A)** or 48 h **(B)**. **(A)** Supernatants were discarded and BMDM were lysed in RLT lysis buffer. Total RNA was extracted as described before and retro-transcribed. *Csf2* expression was determined by quantitative PCR after normalization with three housekeeping genes (*Sdha, Rpl9*, and *Hprt1*). Fold change (FC) data are expressed as the mean ± SD as compared to the mock condition in three independent experiments. Mean values not sharing the same letter are significantly different according to the Student’s *t*-tes*t* (*p* < 0.05). **(B)** After incubation, cell culture supernatants were harvested and stored at −20°C. GM-CSF was measured by ELISA. **(C–E)** WT BMDM were pre-incubated 2 h with rGM-CSF (5 ng/mL) or rM-CSF (5 ng/mL) or medium. **(C)** Cells were further stimulated with 100 µg/mL or 10 µg/mL of BG75 for 8 h. Cell culture supernatants were then removed and TNFα was quantified by ELISA. **(D)** After incubation, cells were harvested to assess surface expression of Dectin-1 and CD11b by flow cytometry. Expression data of Dectin-1 or CD11b are presented as overlaid histograms for each condition. **(E)** After pre-incubation with rGM-CSF, rM-CSF, or medium, BMDM were treated with 100 or 10 µg/mL of BG75 for 8 h before further stimulation with 100 ng/mL of ultraPure LPS or medium for 16 h. Supernatants were collected and TNFα was measured by ELISA. **(F)** BMDM were pre-incubated for 2 h with 10-fold serial dilutions of rGM-CSF from 0.1 to 1000 pg/mL, before assessment as in **(E)**. **(G)** In this experiment, BMDM were first incubated with an anti-GM-CSFR monoclonal antibody or its isotype control for 2 h. rGM-CSF (100 pg/mL) was added to cells for 2 h as a positive control for neutralization. Cells were then stimulated as in **(E)**. Data are expressed as the mean ± SD from two independent experiments performed in triplicate. Mean values not sharing the same letter are significantly different according to the Student’s *t*-test (*p* < 0.05).

We first confirmed by RT-qPCR that, as observed in the microarray analysis, *Csf2* expression was dramatically upregulated in macrophages pretreated with BG and further stimulated by LPS (Figure 4A). This induction appeared highly associated with BG signaling in macrophages, as a huge difference of expression could be observed with BG-enriched preparations and ScCW or mock. At the protein levels, the quantification of GM-CSF (*Csf2* expression product) by ELISA confirmed data from the gene expression analysis and showed an increased production of GM-CSF that correlated with the degree of BG enrichment after a secondary LPS stimulation (Figure 4B). Taken together, these data strongly suggested that GM-CSF production by macrophages was highly associated with BG recognition by macrophages. The use of Dectin-1-deficient macrophages in this model of pretreatment/stimulation induced a strong inhibition of GM-CSF secretion and confirmed that Dectin-1 signaling is essential in this process (Figure 4B). In a previously published study, we demonstrated that *Csf2* expression was readily induced in macrophages stimulated by BG only (23). We also reported that macrophages stimulation with BG resulted in a very marginal secretion of inflammatory cytokines. It has been previously demonstrated that Curd, a linear β(1,3)-glucan from *A. faecalis*, a gram negative bacterium, could synergize with GM-CSF and confer a strong inflammatory signature to dendritic cells (14). Thus, we examined the influence of recombinant GM-CSF on macrophage activation by BG. As previously observed, BG stimulation on its own induced a very low cytokine production (<50 pg/mL) by macrophages cultured in the absence of growth factors (Figure 4C). However, pre-incubating macrophages with rGM-CSF (5 ng/mL) for 2 h before BG stimulation dramatically increased the resulting levels of cytokine secretion. This effect appeared highly associated with rGM-CSF as pre-incubation with rM-CSF (*Csf1* expression product), another major hematopoietic factor influencing macrophages growth, differentiation, and phenotype, did not allow increase of cytokine secretion levels. Using flow cytometry, we confirmed that, as previously described (15), rGM-CSF significantly upregulated the expression of Dectin-1 at the macrophage cell surface (Figure 4D). This effect was not reproduced by pre-incubating cells with rM-CSF. Furthermore, rGM-CSF had no effect on the expression of CR3 (CD11b), another BG receptor. These results suggested that GM-CSF could significantly amplify BG signaling by increasing Dectin-1 expression and consequently be strongly involved in the improved response of BG-primed macrophages to secondary TLR stimuli. And, indeed, pre-incubation of macrophages with rGM-CSF (5 ng/mL) significantly improved this priming and resulted in levels of TNFα secretion higher than the secretion by macrophages pre-incubated with rM-CSF or cultured in the absence of stimulating factors (Figure 4E). Of note, rGM-CSF alone induced a moderate increase of cytokine production upon LPS secondary stimulation. At this step, we could not exclude an endogenous production of GM-CSF during the priming step of macrophages with BG. In a previous study, we showed that macrophage stimulation with BG led to the detection of low levels of GM-CSF secretion (from 1 to 5 pg/mL) (23). Thus, we evaluated the effect of rGM-CSF in a dose-dependent manner using our stimulation model and observed that 10 pg/mL of rGM-CSF were sufficient to improve the priming of macrophages by BG (Figure 4F). As this dose was not very far from the endogenous levels of production previously measured in BG-treated macrophages, we blocked the GM-CSF activity by using a GM-CSFR blocking antibody. For these experiments, we used rGM-CSF (100 pg/mL) to validate blocking conditions. As expected, in the absence of blocking antibody, the pretreatment of macrophages with BG enhanced the response to LPS stimulation and addition of rGM-CSF significantly improved the priming of macrophages by BG (Figure 4G). The addition of anti-GM-CSFR completely abolished this gain of response and confirmed that our experimental conditions allowed blocking the activity of at least 100 pg/mL of GM-CSF. However, the intrinsic ability of BG to prime macrophages response was not affected by the addition of anti-GM-CSFR antibody. This, finally, demonstrated that GM-CSF could improve macrophages priming by BG but was not primarily involved in this mechanism.

### Specific Induction of FHL2 by BG-Enriched Pretreatment Is Dectin-1-Dependent

**Figure 5:**
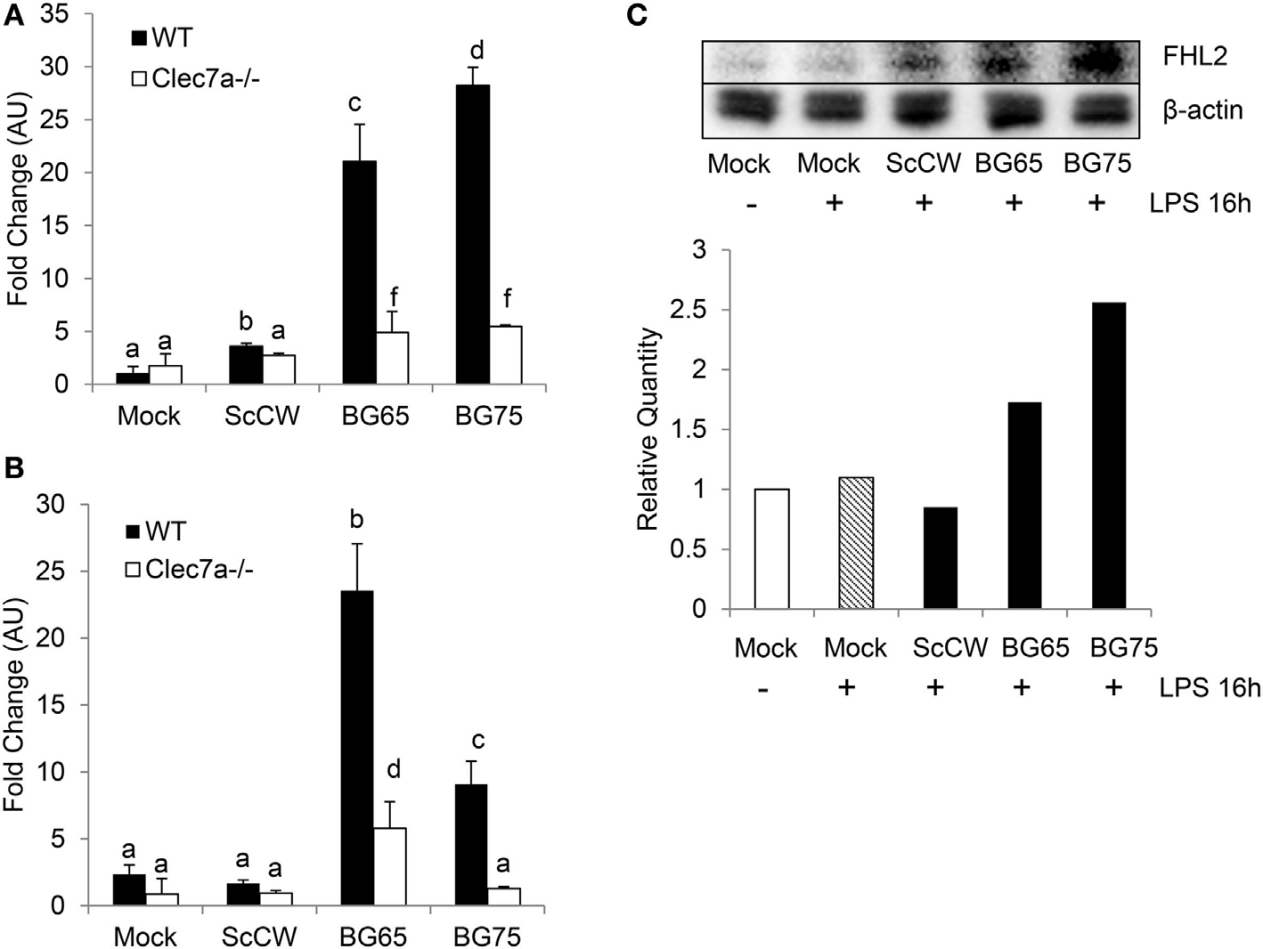
*Fhl2* upregulation induced by BG-enriched compounds is under the control of Dectin-1. **(A)** Wild-type WT or *Clec7a*^−/−^ bone marrow-derived macrophages (BMDM) were pre-treated as described in Figure 4A. *Fhl2* expression was determined by RT-qPCR as described for *Csf2*. **(B)** WT or *Clec7a*^−/−^ BMDM were stimulated with BG-containing compounds for 8 h. At the end of the incubation, BMDM were lysed in RLT lysis buffer. After RNA extraction, cDNA synthesis and RT-qPCR were performed. Data are expressed as the fold change (FC) related to the mock condition value and are representative of two experiments performed in triplicate. **(C)** WT BMDM were stimulated following the same protocol as in **(A)**. After incubation, BMDM were lysed in RIPA buffer supplemented with 5 mM EDTA and protease inhibitor (Pierce™). After clarification and protein quantitation, 10 mg of total lysates were separated on a polyacrylamide gel. After transfer, nitrocellulose membrane was subjected to Western blotting with anti-FHL2 (F4B2-B11) and anti-β-actin (C4) mAbs (Santa Cruz Biotechnologies). Blotting was revealed using chemiluminescence. Results are also presented as the relative quantity of FHL2 protein detected in each condition as compared to the reference corresponding to the unstimulated cells. The data are representative of three independent experiments.

Significant *Fhl2* upregulation in BG-enriched conditions upon a secondary LPS stimulation was confirmed by qPCR (Figure 5A). As *Fhl2* expression seems to be highly correlated with the degree of purity of BG in the preparations we used, we next investigated the influence of Dectin-1 on *Fhl2* transcription following incubation with BG compounds. WT and *Clec7a*^−/−^ BMDM from C57Bl/6 were incubated 8 h with BG65/75 and ScCW to examine *Fhl2* expression. In WT or *Clec7a*^−/−^ cells, the gene was basically poorly expressed and even following treatment with ScCW (Figure 5B). However, induction of *Fhl2* was triggered by BG65 or BG75 pretreatments (around 5-fold upregulations) in WT cells. Interestingly, Dectin-1-deficiency entailed a severe loss of *Fhl2* induction following BG65 treatment and almost abolished it after BG75 incubation. Western Blotting on BMDM lysates confirmed that FHL2 protein was more abundant after pretreatment with BG65 and BG75 (at least 2-fold more as compared to mock) than with ScCW following a secondary LPS stimulation and was poorly detectable in mock condition, with or without LPS stimulation (Figure 5C). Therefore, BG-enriched compounds induce *Fhl2* expression, essentially *via* the Dectin-1 pathway.

## Discussion

As previously described, the stimulation with ScCW or Zym led to a strong inflammatory response by macrophages resulting from a synergy between TLR (mainly TLR2) and Dectin-1-associated signaling (17, 20, 21, 32). By contrast, and as observed in our former study (23), BG-enriched parietal fractions failed to do so. And yet, the experiments presented here suggest that BG exposure profoundly imprints macrophage, phenotype, and functions. Indeed, we demonstrated that, when pretreated with BG-enriched preparations (BG65, BG75, or WGPd), TEPM or BMDM showed an exacerbated cytokine production in response to a secondary TLR-associated stimulus. This priming effect could be revealed through various secondary stimulations, including TLR2 or TLR4 ligands but also live *S. aureus* or *E. coli*. However, whether other ligands or pathogens have the same potential should be further tested.

The pretreatment with soluble BG (WGPs) did not improve macrophage response to LPS stimulation, suggesting that Dectin-1 clustering (33) by particulate BG and subsequent downstream signaling events are prerequisites for macrophage priming. The significant inhibition of priming observed in Dectin-1-deficient macrophages was consistent with this hypothesis. However, this inhibition was only partial and raised the question about the possible involvement of other BG receptors, at least in this experimental model.

Interestingly, the pretreatment with crude ScCW or Zym failed to prime macrophages although both of these yeast-derived preparations do contain BG (15 and 50%, respectively) (23, 34, 35). In a previous study, using TLR pathway reporter cell lines HEK293, we showed that BG enrichment process from ScCW to BG65 and BG75 removed the majority of TLR agonists including mannans (23). Taken together, these observations suggest that the simultaneous targeting of multiple PRRs, leading to a strong inflammatory response of macrophages, prevents the BG-induced priming.

Such a priming effect of BG was previously observed with the pretreatment of human PBMC with the pathogenic yeast *C. albicans* that results in an enhanced response to TLR-associated secondary stimulus (36). The engineering of *C. albicans* strains demonstrated that BG were central in this effect (36). Similarly, pretreating human monocytes with whole *C. albicans* or purified BG not only led to an enhanced cytokine response but also modified the cell shape, granulosity, and expression of surface markers. This “immune training” was observable in short term (24 h) (36) or long-term conditions (6 days) (37). Here, we unambiguously demonstrated that BG could train fully differentiated mouse macrophages in a short-term model. We also evaluated this training for longer resting periods between pretreatment and LPS re-stimulation. For up to 6 days, we observed increased cytokine production by BG-pretreated cells compared to non-pretreated cells (data not shown). However, these experiments were done in the absence of exogenous M-CSF. Under these conditions, non-pretreated macrophages started to present evident signs of cell suffering after 4 days of culture unlike BG-pretreated macrophages that appeared unaffected by growth factor deprivation. At days 5 and 6, cell numbers were clearly decreased in control wells creating an obvious bias in the measurement of cytokine production after LPS re-stimulation. Consequently, we decided to present data up to 3 days after pretreatment, as we did not observe differences in cell numbers that could explain a 5-fold increase in cytokine levels.

Taken together, these results fully demonstrate that exposure to fungal BGs profoundly modifies monocyte/macrophage physiology, and functions. Through a microarray analysis, we showed that BG pretreatment induced a specific set of genes in mouse macrophages. This was highlighted by comparing BG-pretreated cells versus non-pretreated cells or cells pretreated with ScCW that was shown unable to perform training of macrophages. By doing so, we identified molecular pathways preferentially triggered by BG. These pathways were highly related to PI3K cascade upstream of AKT. Consistently, we also found that GPCR signaling, known to activate PI3K cascade, was involved in BG-induced priming of macrophages. These findings were in agreement with previous studies, indicating that BGs induce a metabolic shift toward aerobic glycolysis mediated by an AKT–mTOR–HIF1α pathway in human monocytes (28, 37). Furthermore, BG-trained monocytes revealed higher intracellular ratio of nicotinamide adenine dinucleotide (NAD^+^) to its reduced form (NADH) (28). We showed higher fluorescence of resofurin in BG75-treated BMDM using a Biotool Vita-Blue Cell Viability reagent. This assay is based on a redox reaction that transforms weakly fluorescent resazurin to highly fluorescent resofurin by oxidation of NADH in NAD^+^ in metabolically active cells, meaning that BG-treated BMDM displayed higher NAD^+^/NADH ratio, in line with results reported by *Cheng* et al.

By contrast, ScCW pretreatment mainly induced TLR-associated pathways signaling that is in line with our previous observations, showing a robust inflammatory response of macrophages through NFκB activation mediated by MyD88-dependent pathways after exposure with crude yeast CW or Zym (23). The leukotriene pathway was also triggered by ScCW pretreatment which is consistent with findings concerning Zym activity on human monocytes (38).

The microarray analysis consistently identified *Csf2* (that encodes for GM-CSF), the keystone of the gene network, and *Fhl2* as the two main upregulated genes by BG65 and BG75 priming conditions. IPA analysis revealed their enrichment for inflammatory response, proliferation and differentiation of bone marrow progenitors, migration and binding of cells, in antigen presentation and cell survival, immune cell trafficking, and development of hematological system. These two genes further targeted pathways highlighting the crucial role of cytokines as mediators of the communication between innate and adaptive responses.

GM-CSF is a highly pleiotropic cytokine and a growth factor involved in the differentiation of hematopoietic progenitors from bone marrow but GM-CSF is also recognized as a key mediator during inflammation (39–41).

In this study, we demonstrated that *Csf2* expression and GM-CSF production were highly specific of BG pretreatment as ScCW pretreatment failed to do so. Moreover, this dramatic enhancement was totally abrogated in Dectin-1-deficient macrophages, confirming the strong association between Dectin-1 and GM-CSF production. In agreement with this result, it was demonstrated that pre-incubating dendritic cells with GM-CSF substantially enhances the cytokine secretion upon Curd, a linear BG from *A. faecalis* stimulation (14). By using flow cytometry, we showed that GM-CSF treatment increased Dectin-1 expression on BMDM as previously observed with dendritic cells or with resident and thioglycollate-elicited macrophages treated with GM-CSF or IL-4 or a combination of both cytokines (12, 14, 15). It was, thus, suggested that such an upregulation of Dectin-1 expression consequently to GM-CSF allows Dectin-1-mediated TNFα induction (12). In line with this, GM-CSF was shown to be required to trigger a robust production of TNFα, IL-6, and IL-1β in Curd-stimulated dendritic cells (14).

In a previous study, we evidenced a late Dectin-1-dependent overexpression of *Csf2* following stimulation with BG65 or BG75 but unexpectedly, we found very low amounts (<5 pg/mL) of GM-CSF in culture supernatants even after 24 h of incubation (23). By contrast, the stimulation of peritoneal macrophages with soluble BG from *Grifola frondosa* mushroom results in a significant expression of *Csf2* with subsequent production of GM-CSF (100 pg/mL) after 6 h of incubation (12). However and again in contrast with our results, the induction of GM-CSF production by soluble BG appeared to be Dectin-1-independent confirming that particulate and soluble BG do not trigger similar pathways. In agreement with this, we showed here that, compared to *Sc* particulate BG, soluble BGs (WGPs) were unable to induce macrophages priming.

Our data seem to target GM-CSF as a pivotal actor of the BG-induced priming of macrophages. Consistently with this hypothesis, GM-CSF-treated macrophages/monocytes exposed to TLR2 or TLR4 ligands were shown to produce significantly more IL-1β, IL-6, and TNFα (30, 40–42). Furthermore, GM-CSF was also able to prevent endotoxin tolerance in macrophages by restoring TNFα production on LPS-tolerized human monocytes (43).

Similarly, it was very recently shown that GM-CSF, and IL-3, on its own, was sufficient to train human monocytes in a p38- and SIRT2-dependent manner (30). In this study, we also observed that GM-CSF pretreatment of mouse macrophages induced a moderate but significant increase of cytokine secretion upon LPS re-stimulation. Yet, this training effect remained much lower than the one mediated by BG or GM-CSF plus BG.

As GM-CSF appears to be strongly related to the priming of macrophages by particulate BG, we further investigated the influence of GM-CSF on BG-induced TNFα production.

Using decreasing concentrations of rGM-CSF during BG pretreatment, we showed that 10 pg/mL were enough to improve the priming of macrophages. Taken together with our previous observations, this result suggests that the endogenous production of GM-CSF induced by BG along the pretreatment step could be responsible for macrophage training. To rule out this hypothesis, we inhibited GM-CSF-associated signaling in macrophages by using a GM-CSFR blocking antibody. The amount of blocking antibody used was sufficient to completely inhibit the activity of 100 pg/mL rGM-CSF, a far greater concentration than the one supposed to be produced endogenously following BG pretreatment. Under these conditions, we showed that blockade of GM-CSF pathway did not affect the levels of training induced by BG, highlighting that endogenous GM-CSF is not required for the BG-induced training of macrophages, which clearly argues against results from previous studies in favor of a strong reduction of BG-induced TNFα in macrophages neutralized with anti-GM-CSF antibodies (12, 44).

Taken together, our results demonstrate that GM-CSF, which is endowed with low immune training properties on its own, is not intrinsically involved in the BG-induced training of macrophages although it can substantially improve it. However, we cannot exclude a contribution of GM-CSF in the long-term training model, as it favors cell survival and adherence (39–41, 45). This later hypothesis should be accurately investigated.

Intriguingly, we identified *Fhl2* (four-and-a-half LIM-only protein 2) as the second most upregulated gene in macrophages pretreated with BG and further stimulated with LPS. We demonstrated that *Fhl2* expression was specifically induced by BG in a Dectin-1-dependent manner. At the protein level, we showed that production of FHL2 was significantly increased after BG pretreatment and was not influenced by the secondary LPS stimulation. Here, we revealed for the first time the involvement of *Fhl2* in Dectin-1-mediated pathway. Again, ScCW was not able to modulate FHL2 expression and production, confirming the predominant role of Dectin-1- as compared to TLR-associated pathways.

*Fhl2* is expressed in a cell-and tissue-specific manner and molecular mechanisms by which this protein exerts its roles remain incompletely understood, especially in cells where its expression is limited or absent. Moreover, according to its cytoplasmic or nuclear localization within the cell, FHL2 seems to have highly variable molecular partners as reviewed in Ref. (46) and to play important role as mediator in many signaling pathways, including NFκB signaling pathways (47, 48) or TGF-β signaling (49, 50).

Interestingly, FHL2 was reported to interact with NFκB but in a cell type-dependent manner. Indeed, FHL2 has been shown to inhibit NFκB activity during osteoclastogenesis by displacing TRAF6 from RANK (51). By contrast, FHL2 was shown to mediate IL-6 production through NFκB and p38 MAPK signaling pathway in muscle cells (48). Similarly, FHL2-deficiency in macrophages significantly reduced TNFα and IL-6 production following LPS exposure, suggesting that FHL2 acts as a positive regulator of NFκB through TRAF6 in liver macrophages (47) but also in FHL2-transfected HEK cells (52). Although our study does not bring definitive proofs of FHL2 involvement in short-term training of macrophages, its NFκB activating capacity is not consistent with the high levels of cytokine secretion recorded by BG-primed macrophages upon a secondary TLR-associated stimulation. Indeed, NFκB activity recorded in BG-trained BMDM was similar in ScCW-incubated cells, meaning that BG immune training is poorly correlated with NFκB-related signaling. Consequently, it remains highly plausible that FHL2 interacts with other nuclear transcription factors or cell signaling molecules.

However, there is no evidence that FHL2 activity directly result from Dectin-1 triggering and downstream signaling. Indeed, we observed that at least 8 h of BG pretreatment were needed to induce macrophage priming and that 16 h of incubation with LPS were required to reveal macrophage training. These incubation times are sufficient to allow *de novo* synthesis of molecules that could link Dectin-1 and FHL2 activity. Undeniably, further investigations will be needed to decipher FHL2 regulation by Dectin-1 pathway and the associated molecular mechanisms.

Altogether, we established for the first time a short-term model of trained immunity with mouse macrophages using BG from *S. cerevisiae* and evidenced GM-CSF and FHL2 as potential co-actors of this priming effect. Considering the availability of industrial byproducts of *S. cerevisiae*, reprogramming monocytes/macrophages becomes possible using BG from this source to improve their response against invading pathogens.

## Ethics Statement

WT C57Bl/6 mice were purchased from Janvier Labs (St Berthevin, France) and Clec7a^−/−^ mice (5) were originally provided by Pr. Gordon Brown (University of Aberdeen, Scotland) and were bred in-house. Eight- to 12-week-old C57Bl/6 Clec7a^−/−^ mice and their strain-matched WT controls from both sexes were housed under pathogen-free conditions in an accredited research animal facility of the National Veterinary College (UMR IHAP, Toulouse, France). This study was carried out in strict accordance with the Federation of European Laboratory Animal Science Association guidelines (FELASA). Experiments were performed by FELASA accredited investigators (no. 311155580) and approved by the local ethics committee, “Science et Santé Animale” (SSA). All efforts were made to minimize animal pain and distress.

## Author Contributions

Participated in research design: SW, GF, and GT; conducted experiments: SW, GT, and MF; performed data analysis: SW, GF, and GT; wrote or contributed to the writing of the manuscript: SW, GF, and GT.

## Conflict of Interest Statement

The authors declare that the research was conducted in the absence of any commercial or financial relationships that could be construed as a potential conflict of interest.

## Abbreviations

BG, β-glucan; Curd, curdlan; CW, cell wall; DEG, differentially expressed genes; NEAA, non-essential amino acids; Pam3, Pam3CSK4; PAMP, pathogen-associated molecular pattern; Pen–Strep, penicillin–streptomycin; PRR, pattern recognition receptor; SEAP, secreted embryonic alkaline phosphatase; *Sc, Saccharomyces cerevisiae*; TEPM, thioglycollate-elicited peritoneal macrophages; WGPd, dispersible whole glucan particle; WGPs, soluble whole glucan particle; WT, wild type; Zym, Zymosan.
