# Supplementary material for: Molecular Analysis of a Short-term Model of β-Glucans-Trained Immunity Highlights the Accessory Contribution of GM-CSF in Priming Mouse Macrophages Response
Source: Front Immunol. 2017 Sep 11;8:1089. doi: 10.3389/fimmu.2017.01089 (PMC5601002; doi:10.3389/fimmu.2017.01089)
Supplement: Supplementary file 1 [file data_sheet_1.docx]

Supplementary Material

**Molecular Analysis Of A Short-Term Model Of β-Glucans-Trained Immunity Highlights The Accessory Contribution Of GM-CSF In Priming Mouse Macrophages Response**

Sarah Walachowski^¶^, Guillaume Tabouret^¶^*, Marion Fabre, Gilles Foucras

*** Correspondence:** Dr. Guillaume Tabouret: [g.tabouret@envt.fr](mailto:g.tabouret@envt.fr)

¶ These authors contributed equally to this work.

# Supplementary Figures and Tables

## Supplementary Figures


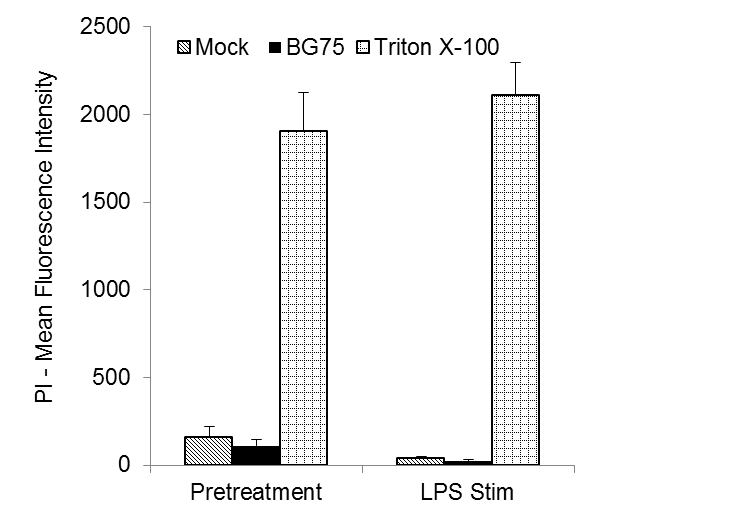


**Figure S1. BMDM pretreatment with BG does not affect cell viability.** BMDM were incubated with BG75 (100 µg/mL) for 8 h and washed three times with warm PBS. At this step, a first viability assessment was conducted by adding propidium iodide (PI) in culture (5µg/mL) and reading the associated fluorescence. A second measurement was performed after incubation of BG75-pretreated BMDM with LPS (100 ng/mL) for 16 h. At each time point, 2.10^5^ control BMDM were incubated for 5 min with Triton X-100 (0,1% final) to determine the maximal level of fluorescence (100% cell lysis). Data are expressed as the mean fluorescence of PI $\pm$SD for each sample.


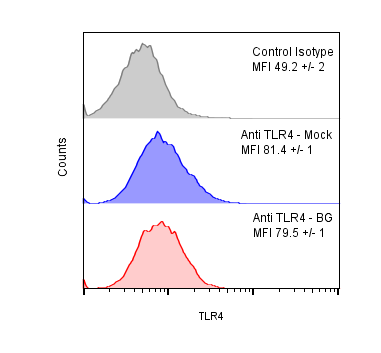


**Figure S2. BMDM pretreatment with BG does not increase TLR4 cell surface.** WT BMDM were incubated with BG (100 µg/mL) for 24 h and surface expression of TLR4 was measured using flow cytometry (Anti-TLR4 monoclonal antibody conjugated with Alexa-488, Clone 267518, Novus Biologicals, France) .


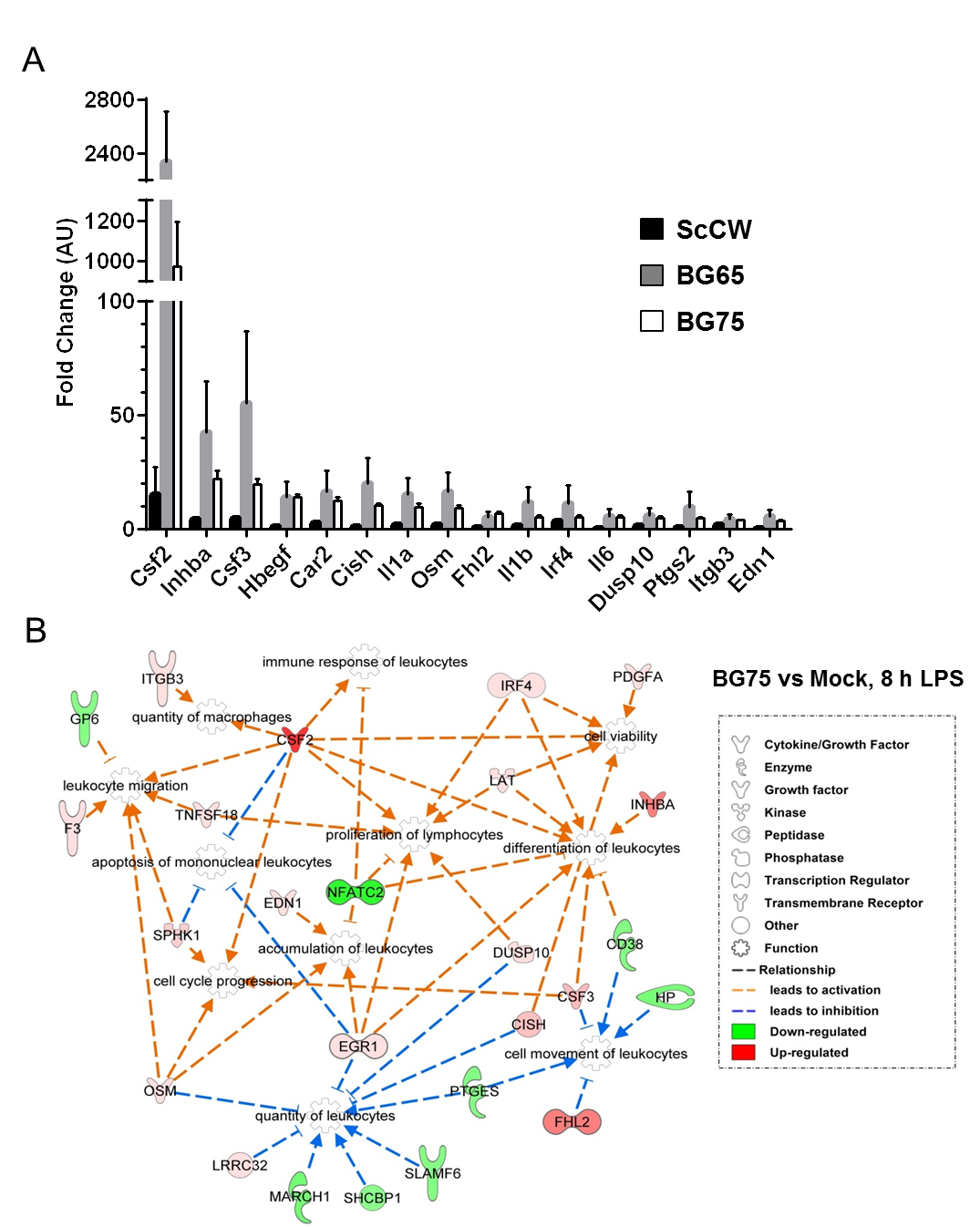


**Figure S3**. **BG-primed BMDM exhibit enrichment for strong inflammatory response and cell viability after LPS exposure.** WT BMDM were handled according to the same procedure as described in Fig 3. After BG pretreatment for 8 h, cells were stimulated with 100 ng/mL of ultraPure LPS for 4 or 8 h. Total RNA was extracted and transcriptomic analysis was performed using Agilent microarrays and GeneSpring software. **(A)** The top-10 list of up-regulated genes in the different BG conditions versus the mock control (*p-value* < 0.001, FC > 1.5) is given. Gene expression was confirmed by RT-qPCR. Data are expressed as the mean $\pm$SD from three independent mice. Comparisons are shown for each BG condition against mock and each mean values from BG65 and BG75 are significantly different from ScCW mean values according to the unpaired two-tailed *Student’s t-test* (*p* < 0.05). **(B)** Using heatmap (presented in Fig 3) of fold-change expression values, from BG-treated BMDM compared to non-treated BMDM upon LPS exposure, analysis of the enriched functions by BG-pretreatment was done (purple clusters 1 and 3: *p-value* < 0.001 and FC > 5) with Ingenuity Pathway Analysis (IPA) software. The results are shown as a network of significant functions associated with the main upregulated genes according to *p-values* (calculated by the Fisher exact test) and z-scores.


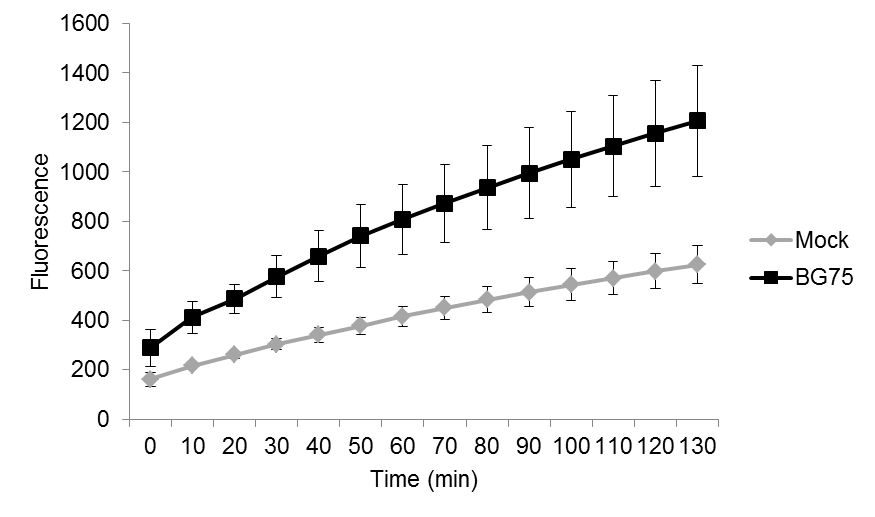


**Figure S4. BMDM pretreatment with BG improves NAD^+^/NADH ratio.** BMDM were stimulated with BG75 for 24 h and resofurin fluorescence (and proportionally NAD^+^/NADH ratio) was measured using Vita-blue cell test (Bimake, Stratech, US) as recommended by the manufacturer. Data are expressed as the mean fluorescence $\pm$SD for each sample at each time points.

## Supplementary Tables

**Table S1: Primers designed for analysis of gene expression in BMDM by quantitative PCR**

|  | Primers (5' -> 3') | |
| --- | --- | --- |
| Genes | **Forward** | **Reverse** |
| *Csf2* | CCGTAGACCCTGCTCGAATA | TGCCTGTCACATTGAATGAA |
| *Fhl2* | TGCTTCTGTGACTTGTACGC | AAGCAGTCGTTGTGCCATTG |
| *Hbegf* | TGTCATCGTGGGACTTCTCATG | ACGCCCAACTTCACTTTCTC |
| *Inha* | ACGGCAAGGTCAACATTTGC | ATAGCCAGAGGGAGCAATGATC |
| *Car2* | ATAAAGCTGCGTCCAAGAGC | TTCAGCACTGCATTGTCCTG |
| *Csf3* | CTGACAGTGACCAGGGGAAC | ATGGCTCAACTTTCTGCCCAG |
| *Cish* | TACTGCAGTGCACCTGAAAC | TGATGACTAGCCGACACAGATG |
| *Osm* | AGACTCCGGCTTTTCCAAAG | GGGCCATGCAGAAAACATTG |
| *Dusp10* | GCCTACTTGATGAAGCACACAC | TTGGTCGTTTGCCTTTGACG |
| *Socs2* | TCCAGATGTGCAAGGATAAACG | AGGTACAGGTGAACAGTCCCATT |
| *Itgb3* | ACGGATACTGGCAAAAACGC | ACTGGAATCTGACGACACAGTC |
| *Edn1* | ACATCATCTGGGTCAACACTCC | TTTTTGGTGAGCGCACTGAC |
| *Il1a* | AGATGACCTGCAGTCCATAACC | TGACAAACTTCTGCCTGACG |
| *Irf4* | AATGTCCTGTGACGTTTGGC | TGGGGCACAAGCATAAAAGG |
| *Ptgs2* | AGAACCTGCAGTTTGCTGTG | ATGTCGCACACTCTGTTGTG |
| *Il6* | GAGGATACCACTCCCAACAGACC | AAGTGCATCATCGTTGTTCATACA |
| *Il23a* | TGTGCCCCGTATCCAGTGT | CGGATCCTTTGCAAGCAGAA |
| *Ccl2* | CTTCTGGGCCTGCTGTTCA | CCAGCCTACTCATTGGGATCA |
| *Il1b* | CAACCAACAAGTGATATTCTCCATG | GATCCACACTCTCCAGCTGCA |
| *Il10* | GGTTGCCAAGCCTTATCGGA | ACCTGCTCCACTGCCTTGCT |
| *Tnfa* | CATCTTCTCAAAATTCGAGTGACAA | TGGGAGTAGACAAGGTACAACCC |
| *Ccl24* | TGTGGGTTCAGAGGCACATAC | ACTTGGTTCTCACTGCCTTG |
| *Mrc1* | ATGCCAAGTGGGAAAATCTG | TGTAGCAGTGGCCTGCATAG |
| *Cx3cr1* | TGCCCTTGCTTATCATGAGC | TGGCCTTCTTGCGATTCTTG |
| *Ch25h* | ACAAAATGCTGGGCACTCTG | ATCAAGTGTACAGCGCATCG |
| *Nos2* | ACATCCTGCAAAAGCAGCTG | AGCTTCTTCAACGTGGTAGC |
| *Sdha* | ATTGTGCCTGGTCTGTATGC | AATTTGCTCCAAGCCGGTTG |
| *Rpl9* | TGGTCCCTGCTCTCAAG | GGCCTTTTCCTTCCGTTTCTC |
| *Hprt1* | AGGACTGAAAGACTTGCTCGAG | AATCCAGCAGGTCAGCAAAG |

**Table S2:** **Top-10 list of the most down-regulated genes after BG75 treatment upon LPS exposure**

| ↓Down-regulated, Pretreated vs mock (FC) | | | 4 h LPS | | | 8 h LPS | | |
| --- | --- | --- | --- | --- | --- | --- | --- | --- |
| Rank | **Gene** | **Gene Title** | **ScCW** | **BG65** | **BG75** | **ScCW** | **BG65** | **BG75** |
| 1 | *Cx3cr1* | Chemokine (C-X3-C) receptor 1 | -2.6 | -2.6 | -2.1 | -6.8 | -5.8 | **-3.9** |
| 2 | *Pcp4l1* | Purkinje cell protein 4-like 1 | -9.7 | -2.4 | -2.6 | -10.2 | -2.8 | **-3.1** |
| 3 | *Ccl24* | Chemokine (C-C motif) ligand 24 | -3.6 | -2.5 | -2.6 | -4.7 | -3.0 | **-3.0** |
| 4 | *Slc9a9* | Solute carrier family 9 member 9 | -3.5 | -2.5 | -2.4 | -2.8 | -1.9 | **-3.0** |
| 5 | *Net1* | Neuroepithelial cell transforming gene 1 | -1.9 | -1.2 | -1.2 | -4.3 | -2.8 | **-2.8** |
| 6 | *Ramp1* | Receptor activity modifying protein 1 | -3.2 | -2.4 | -2.5 | -3.1 | -2.5 | **-2.7** |
| 7 | *Tmem114* | Transmembrane protein 114 (Cldn26) | 1.0 | 1.6 | 1.3 | -3.0 | -2.7 | **-2.6** |
| 8 | *Nudt17* | Nudix-type motif 17 | -4.2 | -3.4 | -2.7 | -3.6 | -1.9 | **-2.6** |
| 9 | *Stab2* | Stabilin 2 | -6.1 | -2.4 | -2.4 | -2.7 | -1.5 | **-2.5** |
| 10 | *Acat3* | Acetyl-Coenzyme A acetyltransferase 3 | -2.4 | -2.5 | -2.3 | -3.0 | -2.9 | **-2.4** |
